# Protein sequences classification by means of feature extraction with substitution matrices

**DOI:** 10.1186/1471-2105-11-175

**Published:** 2010-04-08

**Authors:** Rabie Saidi, Mondher Maddouri, Engelbert Mephu Nguifo

**Affiliations:** 1 LIMOS - Blaise Pascal University - Clermont University, BP 10448, Clermont-Ferrand 63000, France; 2 LIMOS - CNRS UMR 6158, Aubière 63173, France; 3 Department of Computer Science - FSJ - University of Jendouba, UMA Street, Jendouba 8100, Tunisia; 4 URPAH - FST - University of Tunis El Manar, Academic Campus, Tunis 2092, Tunisia; 5 Department of Computer Science - FSG - University of Gafsa, Campus of Sidi Ahmed Zarroug, Gafsa 2112, Tunisia

## Abstract

**Background:**

This paper deals with the preprocessing of protein sequences for supervised classification. Motif extraction is one way to address that task. It has been largely used to encode biological sequences into feature vectors to enable using well-known machine-learning classifiers which require this format. However, designing a suitable feature space, for a set of proteins, is not a trivial task. For this purpose, we propose a novel encoding method that uses amino-acid substitution matrices to define similarity between motifs during the extraction step.

**Results:**

In order to demonstrate the efficiency of such approach, we compare several encoding methods using some machine learning classifiers. The experimental results showed that our encoding method outperforms other ones in terms of classification accuracy and number of generated attributes. We also compared the classifiers in term of accuracy. Results indicated that SVM generally outperforms the other classifiers with any encoding method. We showed that SVM, coupled with our encoding method, can be an efficient protein classification system. In addition, we studied the effect of the substitution matrices variation on the quality of our method and hence on the classification quality. We noticed that our method enables good classification accuracies with all the substitution matrices and that the variances of the obtained accuracies using various substitution matrices are slight. However, the number of generated features varies from a substitution matrix to another. Furthermore, the use of already published datasets allowed us to carry out a comparison with several related works.

**Conclusions:**

The outcomes of our comparative experiments confirm the efficiency of our encoding method to represent protein sequences in classification tasks.

## Background

Analysis and interpretation of biological sequence data is a fundamental task in bioinformatics. Classification and prediction techniques are one way to deal with such task [1]. In fact, biologists are often interested in identifying the family to which a lately sequenced protein belongs [2]. This makes it possible to study the evolution of this protein and to discover its biological functions. Furthermore, the study and the prediction of oligomeric proteins (quaternary structures) are very useful in biology and medicine for many reasons [3]. Indeed, they often intervene in terms of bio-macromolecules functional evolution, reparation of misfolds and defects [4,5]. They are also involved in many important biological processes such as chromosome replication, signal transduction, folding pathway and metabolism [6]. Biologists also seek, for instance, to identify active sites in proteins and enzymes [7], to classify parts of DNA sequences into coding or non-coding zones or to determine the function of the nucleic sequences such as the identification of the promoter sites and the junction sites [8-10].

Alignment is the main technique used by biologists to look for homology among sequences, and hence to classify new sequences into already known families/classes. Since relevant information is represented by strings of characters, this technique generally doesn't enable the use of well-known classification techniques such as decision trees (DT), naïve bayes (NB), support vector machines (SVM) and nearest neighbour (NN) which have proved to be very efficient in real data mining tasks [11]. In fact, those classifiers rely on data described in a relational format.

Meanwhile, different studies have been devoted to motif extraction in biological sequences [12-17]. Motifs extraction methods are generally based on the assumption that the significant regions are better preserved during the evolution because of their importance in terms of structure and/or function of the molecule [13], and thus that they appear more frequently than it is expected.

**Figure 1:**
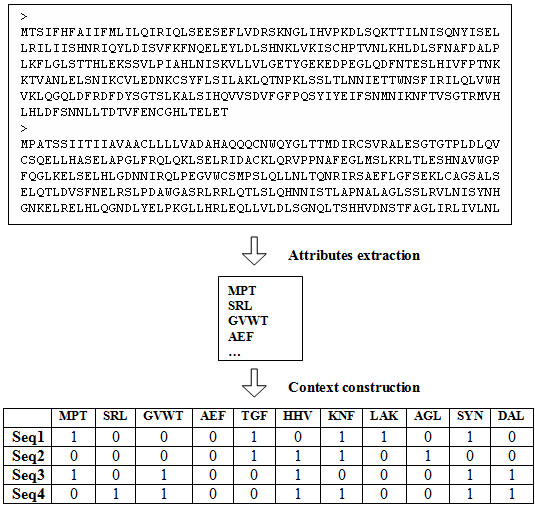
**Sequence pre-processing based on motif extraction**. This figure describes the process of sequence encoding. The extracted motifs are used as attributes to build a binary context where each row represents a sequence.

In [14], authors have shown that motif extraction methods can efficiently contribute to the use of machine learning algorithms for the classification of biological sequences. In this case, the classification obeys the *knowledge discovery in data *(KDD) process and hence comprises three major steps:

1. Preprocessing consists of extracting motifs from a set of sequences. These motifs will be used as attributes/features to construct a binary table where each row corresponds to sequence. The presence or the absence of an attribute in a sequence is respectively denoted by 1 or 0. This binary table is called a *learning context*. It represents the result of the preprocessing step and the new sequence encoding format (figure 1).

2. In the mining step, a classifier is applied to the learning context to generate a classification model.

3. The latter model is used to classify other sequences in the post-processing step. These sequences are also encoded into a relational format using the same features as for the learning context i.e., *test context*.

In a previous work [18], we proposed a new method to encode protein sequences. It extends an existing method, termed Discriminative Descriptors (DD) [14], by taking into account the fact that some amino acids have similar properties and thus can be substituted by each other while changing neither the structure nor the function of the protein [19]. Hence, there might be several motifs that could be replaced by a single motif. We used amino acids substitution matrices to define such similarity; our encoding method is termed Discriminative Descriptors with Substitution Matrix (DDSM). Preliminary experiments conducted with C4.5 decision tree have shown promising results [18]. This manuscript presents a detailed experimental comparison (in terms of classification accuracy and number of attributes) between several encoding methods using various kinds of classifiers (C4.5 decision tree, NB, SVM and NN) as well as the standard approach based on alignment using Blast [20].

## Methods and Results

### Some existing feature construction methods

The following is a presentation of five existing methods of features construction: the N-Grams (NG), the Active Motifs (AM), the Amino Acid Composition (AAC), the Functional Domain Composition (FDC) and the Discriminative Descriptors (DD). After this, we re-describe our approach which consists of modifying the DD method by the use of a substitution matrix (DDSM) [18].

#### N-Grams

The simplest approach is that of the N-Grams, known also as N-Words or length N fenestration [21]. The motifs to be built have a predefined length. The N-gram is a subsequence composed of N characters, extracted from a larger sequence. For a given sequence, the set of the N-grams which can be generated is obtained by sliding a window of N characters on the whole sequence. This movement is carried out character by character. With each movement a subsequence of N characters is extracted. This process is repeated for all the analyzed sequences. Then, only the distinct N-grams are kept.

#### Active Motifs

This method allows extracting the commonly occurring motifs whose lengths are longer than a specified length, called Active Motifs, in a set of biological sequences. The activity of a motif is the number of matching sequences given an allowed number of mutations [22]. The motif extraction is based on the construction of a Generalized Suffix Tree (GST) which is an extension of the suffix tree [23] and is dedicated to represent a set of n sequences indexed each one by i = 1..n.

#### Amino Acid Composition

According to the classic definition of this method, the feature set consists of 20 components, representing the 20 native amino acids in proteins. The amino acid composition refers to the occurrence frequency of each of these 20 components in a given protein. Since the information in the primary sequence is greatly reduced by considering the amino acid composition alone, other considerations have been taken into account within several studies such as the sequence-order correlation factors i.e., new features were added to the 20 original which yielded several AAC variants [24-28].

#### Functional Domain Composition

Biological databases, such as PFAM [29] and ASTRAL, contain large collections of multiple sequence alignments and Hidden Markov Model (HMM) profiles covering many common protein domains and families [29]. Functional domains are determined using computational means, especially HMM profiles, combined with biologist knowledge and other databases information. Since they allow variable length gaps between several components, where each component is a simple motif [15,16], functional domains can be considered as structured motifs. But they are more reliable since they obey the expert assessment.

#### Descriminative Descriptors

Given a set of n sequences, assigned to P families/classes F_1_, F_2 _.., F_P_, this method consists of building substrings called Discriminative Descriptors DD which allow to discriminate a family F_i _from other families F_j_, with i = 1..P and i ≠ j [14].

This method is based on an adaptation of the Karp, Miller and Rosenberg (KMR) algorithm [30]. This algorithm identifies the repeats in character strings, trees or tables. The extracted repeats are then filtered in order to keep only the discriminative and minimal ones.

A substring X is considered to be discriminative between the family F_i _and the other families F_j_, with i = 1..P, j = 1..P and i ≠ j if:

1. 

2. 

where α and β are user-specified thresholds between 0 and 1.

### Proposed method: Discriminative Descriptors with Substitution Matrix

In the case of protein, the Discriminative Descriptors method neglects the fact that some amino acids have similar properties and that they can be therefore substituted by each other while changing neither the structure nor the function of the protein [19]. Indeed, we can find several motifs in the set of the attributes generated by the DD method, which are similar and can derive all from a single motif. In the same way, during the construction of the context (binary table), we are likely to lose information when we denote by 0 the absence of a motif while another one, that can replace it, already exists [18].

As mentioned, the similarity between motifs is based on the similarity between the amino acids which constitute them. Indeed, there are various degrees of similarity between amino acids. Since there are 20 amino acids, the mutations between them are scored by a 20 × 20 matrix called a substitution matrix [19,21,31].

#### Terminology

Let  be a set of n motifs, denoted each one by  [p], p = 1.. n.  can be divided into *m *clusters. Each cluster contains a main motif M* and probably other motifs which can be substituted by M*. The main motif is the one which has the highest probability of mutating to another in its cluster. For a motif *M *of *k *amino acids, this probability, noted *P*_*m*_*(M)*, is based on the probability *P*_*i*_*(i = 1.. k) *that each amino acid *M [i] *of the motif *M *does not mutate to any other amino acid. We have:

*P*_*i *_is calculated based on the substitution matrix according to the following formula:

*S(x, y) *is the substitution score of the amino acid y by the amino acid x as it appears in the substitution matrix. *S*^+^*(x, y) *indicates a positive substitution score. *AA*_*j *_is the amino acid of index *j *among the 20 amino acids.

For our purposes, a motif *M *substitutes a motif *M' *if:

1. *M *and *M' *have the same length *k*,

2. *S(M [i], M' [i]) *> = 0, *i *= 1 .. *k*,

3. *SP(M, M') *> = *T*, where *T *is a user-specified threshold such that 0 < = *T *< = 1.

We denote by *SP*(*M*, *M'*) the substitution probability of the motif *M' *by the motif *M *having the same length *k*. It measures the possibility that *M *mutates to *M'*:

*S*_*m *_(*X*, *Y*) is the substitution score of the motif *Y *by the motif *X*. It is computed according to the following formula:

It is clear, according to any substitution matrix, that there is only one best motif which can substitute a motif *M*. It is obviously itself, since the amino acids which constitute it are better substituted by themselves. This proves that the substitution probability of a motif by another one, if they satisfy the substitution conditions, will be between 0 and 1.

#### Methodology

The encoding method is composed to two parts. First, the number of extracted motifs will obviously be reduced because we will keep only one motif for each cluster of substitutable motifs of the same length. Second, we will modify the context construction rule. Indeed, we will denote by 1 the presence of a motif or of one of its substitutes. The first part can be also divided into two phases: (1) identifying clusters' main motifs and (2) filtering. (1) The main motif of a cluster is the one that is the most likely to mutate to another in its cluster. To identify all the main motifs, we sort  in a descending order by motif lengths, and then by *P*_*m*_. For each motif *M' *of , we look for the motif *M *which can substitute *M' *and that has the highest *P*_*m *_(probability of mutation to another motif). The clustering is based on the computing of the substitution probability between motifs. We can find a motif which belongs to more than one cluster. In this case, it must be the main motif of one of them. (2) The filtering consists of keeping only the main motifs and removing all the other substitutable ones. The result is a smaller set of motifs which can represent the same information as the initial set.

#### Example

**Figure 2:**
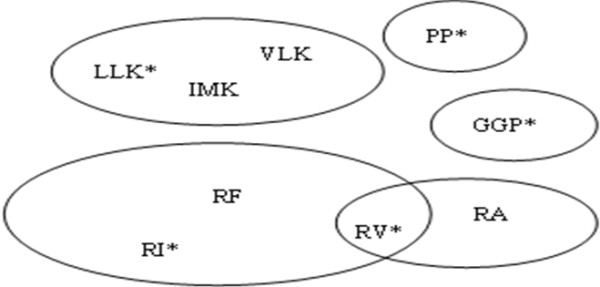
**Motifs clustering**. This figure illustrates the set of clusters and main motifs obtained from the data of table 1 after application of our algorithm. RV belongs to 2 clusters and is the main motif of one of them.

Given a Blosum62 substitution matrix and the following set of motifs (table 1) sorted by their lengths and *P*_*m*_, we assign each motif to a cluster represented by its main motif. We get 5 clusters illustrated by the diagram shown in figure 2.

**Table 1:** Motifs clustering

	LLK	IMK	VMK	GGP	RI	RV	RF	RA	PP
*P*_*m*_	0.89	0.87	0.86	0	0.75	0.72	0.72	0.5	0
Main motif	LLK	LLK	LLK	GGP	RI	RI	RI	RV	PP

is a set of motifs (table 1) sorted by their lengths and *P*_*m*_. The third row shows the cluster main motifs.

### Experimental environment

NG, AM, DD and DDSM encoding methods are implemented in C language and gathered into a DLL library (additional file 1). The accepted format of the input files is the FASTA format for biological sequences files. The library code that we have implemented generates relational files under various formats such as the ARFF format used by the workbench WEKA [32] and the DAT format used by the system DisClass [14].

Our experiments are divided into 2 parts. In the first one, we make a detailed comparison between NG, AM, DD and DDSM encoding methods to confirm the results obtained in [18]. We perform the sequence classification using DT, SVM, NB and NN algorithms as described in section 1. We also conducted classification experiments using Blast [20] i.e., we assign to a protein query the class with the best hit score. Our method (DDSM) constructs the features using the substitution matrix Blosum62. The choice of this substitution matrix is not based on preliminary experiments, but instead on the fact that it is the most used by alignment tools especially the widespread Blast. We examine three aspects:

1. The effect of each encoding method on the four classifiers to deduce which one is the best in terms of accuracy and number of generated attributes.

2. The comparison of the four classifiers while varying the encoding methods.

3. The comparison with Blast results.

In the second part, we try to assess the effect of varying the substitution matrices on our method and on the classification quality and hence to determine whether there is a substitution matrix which could be recommended. Then we compare our feature-construction method with other ones presented in [27,28,33], which means that we compare with nine related works [27,28,33-39].

#### Part 1

To perform our experiments, we use 5 datasets comprising 1604 protein sequences from Swiss-Prot [40] and SCOP [41] described in table 2 (additional file 2).

**Table 2:** Experimental data

Dataset (source)	Identity percentage (%)	Family/class	Size	Total
DS1 (Swiss-prot)	48	High-potential Iron-Sulfur Protein	19	60
		Hydrogenase Nickel Incorporation Protein HypA	20	
		Hlycine Dehydrogenase	21	

DS2 (Swiss-prot)	48	Chemokine	255	510

		Melanocortin	255	

DS3 (Swiss-prot)	25	Monomer	208	717
		Homodimer	335	
		Homotrimer	40	
		Homotetramer	95	
		Homopentamer	11	
		Homohexamer	23	
		Homooctamer	5	

DS4 (Swiss-prot)	28	human TLR	14	40
		Non-human TLR	26	

DS5 (SCOP)	84	All-α domain	70	277
		All-β domain	61	
		α/β domain	81	
		α + β domain	65	

We try to conduct our experiments on various kinds of datasets. These datasets differ from one another in terms of size, number of class, class distribution, complexity and sequence identity percentage. The first dataset DS1 contains 3 distinct and distant protein families. We suppose that classification in this case will be relatively easy since each family will probably have preserved patterns which are different from those of other families [13]. DS2 represents a bigger dataset comprising two sub-families of protein sequences belonging to the *Rhodopsin Like/Peptide *family. However, the datasets DS3 and DS4 present more difficult classification problems. DS3 contains seven classes that represent seven categories of quaternary (4D) protein structure with a sequence identity of 25%. The problem here lies in recognizing the 4D structure category from the primary structure. In this case, an important question is to be answered: does the primary structure contain sufficient information to identify the 4D structure? The task relative to DS4 is that of distinguishing between the human *Toll-like Receptors *(TLR) protein sequences and the non-human ones. The difficulty is due to the structural and functional similarity of the two groups. The choice of this dataset came after Biologists of Pasteur Institute of Tunis (PIT) asked to help them in identifying TLR families especially human ones among the 40 TLR that exist. DS5 consists of 277 domains: 70 all-α domains, 61 all-β domains, 81 α/β domains, and 65 α+β domains from SCOP [41]. This challenging dataset was constructed by Zhou [28] and has been extensively used to address structural class prediction [27,28,34-39].

#### Part 2

In this part, we consider again the datasets DS3, DS4 and DS5 since they are considered to be delicate classification tasks and can thus reveal valuable information about the efficiency of the classifiers and the feature-construction methods. We try to investigate the effect of the substitution matrices variation on the quality of our encoding method and hence on the classification quality using C4.5, SVM, NB and NN algorithms. We employ all the substitution matrices used by the standalone version of Blast and belonging to the two well-known families: Blosum [19] and Pam [42] i.e., Blosum45, Blosum62 Blosum80, Pam30, Pam70, Pam 250.

Since DS3 is the same dataset as in [33], these experiments allow us to compare our encoding method with other related ones presented in that paper, where the nearest neighbour algorithm NN was coupled with each of the following methods: functional domain composition FDC, amino acid composition AAC and Blast alignment tool [20], to predict the quaternary structures categories of the proteins. In fact, the investigation of the quaternary structures prediction using computational tools remains a task with important implications for many reasons. First, these structures are involved in many biological processes and have direct link with known diseases like sickle-cell anaemia. Second, the in vitro methods are very slow and costly in spite of being accurate. This comparison allows us to assess whether our feature-construction method could offer any benefits over the above-mentioned methods quoted in [33] while using the same classifier (NN) and learning technique (leave-one-out).

Since prior information on the structure of a protein can provide useful information about its function, many other works similar to [33] have investigated this topic [27,28,34-39,43-46]. These works often use kinds of amino acid composition or functional domain composition to deal with the prediction of oligomeric proteins or protein structural classes. DS5 represents a challenging dataset that has been extensively used to address structural class prediction [27]. This allows us to compare our method with several works existing in the literature.

## Discussion and Conclusions

### Experimental Techniques

The computations are carried out on a computer with an Intel Centrino 1.6 GHz CPU and 1Go of main memory. Results are shown in the next sub-sections tables. Best accuracies, for each dataset, are shown in bold and results below minimum accepted values results are underlined. The minimum accepted value (MAV) is obtained by assigning all the sequences of a dataset to its biggest class. Hence, we have 35%, 50%, 46.7%, 65% and 29.2% as MAVs respectively for DS1, DS2, DS3, DS4 and DS5. We also show the number of attributes generated by each method.

In the classification process, we use the leave-one-out technique [11] also known as *jack-knife test*. For each dataset (comprising n instances), only one instance is kept for the test and the remaining part is used for the training. This action is repeated n times. The leave-one-out is considered to be the most objective test technique compared to the other ones i.e., *hold-out, n-cross-validation*. Indeed the leave-one-out test allows to obtain the same classification results regardless of the number of runs, which is not the case for the other tests (see the monograph [47] for the mathematical principle and [48] for a comprehensive discussion). For the encoding methods, we use default parameters as in [18]: NG (N = 3), AM (min-length = 3, activity = 25%), DD and DDSM (α = 0, β = 0 except for DS3 where β = 1 to reduce the runtime), DDSM (substitution matrix = Blosum62, substitution probability threshold T = 0.9). These parameters can also be specified by users.

We recall that in part 1, we use the following classifiers: C4.5 decision tree, support vector machine SVM, naïve bayes NB and nearest neighbour algorithm NN of the workbench WEKA [32]. We generate and test the classification models; then we report the classification accuracy (rate of correctly classified sequences). Moreover, we conducted the leave-one-out test on the same datasets using Blast as already explained in section 2.3. In part 2, we investigate any potential effect of the substitution matrix variance on the features building and the classification quality, and then we compare it with other classification systems quoted in [27,28,33].

### Part 1 Results

The experimental results vary according to the input data (table 3 and table 4). The classification of the datasets DS1 and DS2 was relatively easy, as expected. Each family probably has its own motifs which characterize it and distinguish it from the others. This explains the high accuracies reached by all the classifiers with all the encoding methods. But it is notable that the N-Grams encoding gave the best results although it is the simplest method to use. Moreover, since this kind of classification, is easy, it does not require any sophisticated preprocessing and can simply be addressed by using alignment tools; indeed Blast arrived at full accuracy (table 4).

**Table 3:** Machine learning classifiers coupled with encoding methods

			Encoding method			
**Data**	**Mtr**	**Clfr**	**NG**	**AM**	**DD**	**DDSM**

DS1	CA	C4.5	96.7	95	95	96.7
		SVM	**96.7**	93.3	**96.7**	**96.7**
		NB	86.7	90	81.7	80
		NN	63.3	78.3	60	61.7
	
	NA		4935	2060	4905	2565
	
DS2	CA	C4.5	99.6	99.4	99.8	99.4
		SVM	**100**	99.4	**100**	**100**
		NB	**100**	74.7	**100**	**100**
		NN	**100**	**100**	**100**	98.8
	
	NA		6503	7055	10058	1312
	
DS3	CA	C4.5	36.4	-	36.7	**79.2**
		SVM	43.2	-	43.2	78.94
		NB	43.2	-	43.1	59.4
		NN	20.9	-	21.3	77
	
	NA		7983	-	8403	508

DS4	CA	C4.5	60	57.5	77.5	82.5
		SVM	67.5	65	87.5	87.5
		NB	57.5	40	92.6	**95**
		NN	52.5	60	80	80
	
	NA		5561	3602	7116	5505

DS5	CA	C4.5	75.5	75.1	67.9	73.3
		SVM	84.1	81.2	82.3	82.3
		NB	77.3	63.7	84.5	**85.9**
		NN	80.5	79.4	78	78
	
	NA		6465	2393	13830	13083

Mtr: Metric, Clfr: Classifier, CA: Classification Accuracy (%), NA: Number of Attributes.

**Table 4:** Comparison between Blast and DDSM in term of accuracy (%)

Dataset	Blast-based	(DDSM & SVM)	Best of DDSM (from table 3)
DS1	100	96.7	96.7
DS2	100	100	100
DS3	69.60	78.94	79.2
DS4	78.57	87.5	95
DS5	78.3	82.3	85.9

**Figure 3:**
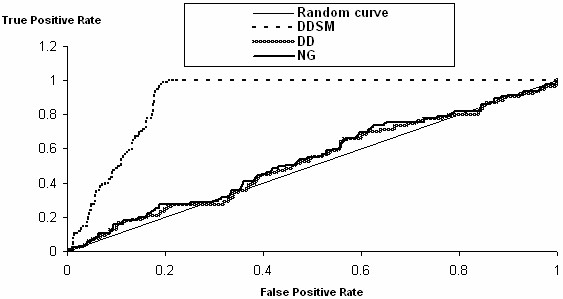
**ROC curve samples for the NB classifier in the dataset DS3 with the DDSM, DD and NG encoding methods**. The positive class is Homotetramer. This figure shows a sample of ROC curves of the NB classifier based on the DDSM, DD and NG encoding methods with Homotetramer as the positive class (DS3). It appears that the DDSM based ROC curve is obviously higher than the two other ones. A ROC graph enables to compare two or more supervised learning algorithms. It depicts relative trade-offs between true positive rates and false positive rates [49]. It is possible to derive a synthetic indicator from the ROC curve, known as the AUC (Area Under Curve - Area Under the Curve). The AUC indicates the probability that the classifier will rank a randomly chosen positive instance higher than a randomly chosen negative instance. There exists a threshold value: if we classify the instances at random, the AUC will be equal to 0.5, so a significant AUC must be superior to this threshold.

As for DS3, classification represents a real challenge. In fact, it is comprised of 717 sequences unequally distributed into seven classes which represent seven quaternary protein structure categories. It is a question of predicting the 4D structure based only on the primary structure without any complementary information. The AM method could not be used because it generates a great number of attributes (dashes in table 3). The obtained accuracies with the NG and the DD methods were below the MAV (within 20.9% and 43.2%) and the result obtained by Blast was acceptable (69.60%) while the best accuracy reached (79.2%) was obtained with the DDSM method (figure 3 illustrates a sample of ROC curves [49] of the NB classifier based on the DDSM, DD and NG encoding methods with Homotetramer as the positive class from DS3).

The dataset DS4 was not as easy to classify as DS1 and DS2 since the human TLR and the non-human TLR resemble each other in terms of function and structure. Indeed the two classes share many similar parts, making it difficult to discriminate them. That is why alignment based classification (using Blast) didn't reach full accuracy as it did for the two first datasets. The NG and the AM encoding seems to be inefficient since they gave accuracies below the MAV with two classifiers. The DD method outperforms the two previous methods (NG and AM). Since it adopts a discriminating approach to build the attributes, it allowed a better distinction between the human TLR and the non-human TLR. But, to improve classification in the dataset DS4, it is necessary to take into account the phenomenon of mutation and substitution between the amino acids which constitute the protein sequences. Indeed, the DDSM method made it possible to reach the highest precisions with all the classifiers, while reducing the number of generated attributes. Experimental results obtained with DS5 show a good performance for all the encoding methods, though no full accuracy was reached. We can notice that NG performed very well and allowed to improve results with the classifiers C4.5, SVM and NN. Blast allowed also to obtain good accuracy which is due to the high identity percentage within the dataset. But, the best accuracy was obtained with DDSM (≈ 86%).

Moreover, we can notice that SVM generally provided the best accuracies with all the encoding methods, though it is known as a slow classifier. So, we can conclude that the combination (DDSM, SVM) could be an efficient system for the protein sequences classification.

### Part 2 results

In this section, we study the effect of the substitution matrices (SM) variation on the classification by applying some of the most often used SMs belonging to the two well-known families: Blosum and Pam [19,42]. These SMs are the same used by the standalone version of Blast [20].

Substitution scoring is based on the substitution frequencies seen in multiple sequence alignments. Yet it differs from Pam to Blosum. Whereas the Pam matrices have been developed from global alignments of closely related proteins, the Blosum matrices are based on local multiple alignments of more distantly related sequences. This would have an effect on the representation size. Indeed, the number of constructed features varies from a substitution matrix to another. Blosum matrices with low numbers and Pam matrices with higher numbers allow the building of fewer features since they score highly the substitution between amino acids. This would yield larger clusters of substitutable motifs, and hence fewer main motifs i.e., fewer features (see section 2.2.2 and 2.2.3).

However, the variances of accuracies are slight when varying the substitution matrices with the same classifier (table 5, table 6 and table 7). Moreover, no substitution matrix allows obtaining the best accuracy for all the classifiers. We can even notice contradicting results; indeed, in DS3 and DS4, NN algorithm performs worse when coupled with Pam30, while the same matrix allows SVM to reach its best accuracy. The same phenomenon is noticed in DS5 with the classifiers C4.5 and SVM and the matrix Pam250. If one looks for reduced-size representation, Blosum matrices with low numbers and Pam matrices with higher numbers are recommended.

**Table 5:** Experimental results per substitution matrix for DS3

Substitution matrix	Attributes	Accuracy (%)			
		
		C4.5	SVM	NB	NN
Blosum45	377	78.5	79.2	59.4	77.7
Blosum62	508	79.2	78.9	59.4	77
Blosum80	532	77.6	80.5	60	77.6
Pam30	2873	77.8	82	60.3	76.7
Pam70	802	78.1	80.5	60.5	77
Pam250	1123	77.3	79.4	59.6	78.7

**Table 6:** Experimental results per substitution matrix for DS4

Substitution matrix	Attributes	Accuracy (%)			
		
		C4.5	SVM	NB	NN
Blosum45	5095	82.5	85	95	80
Blosum62	5505	82.5	87.5	95	80
Blosum80	5968	72.5	87.5	92.5	80
Pam30	7005	82.5	92.5	92.5	65
Pam70	5846	82.5	85	92.5	80
Pam250	1948	82.5	77.5	95	80

**Table 7:** Experimental results per substitution matrix for DS5

Substitution matrix	Attributes	Accuracy (%)			
		
		C4.5	SVM	NB	NN
Blosum45	12603	69.3	82.3	85.9	78
Blosum62	13083	73.3	82.3	85.9	78
Blosum80	13146	70.1	82.3	84.1	78
Pam30	13830	69.3	82.3	84.5	78
Pam70	13822	70.4	82.3	84.5	78
Pam250	1969	66.1	85.2	79.4	78

Since we used the same dataset (DS3) and the same assessment technique (leave-one-out) as in [33], we compare our feature-building method (DDSM with default parameter values: α = 0, β = 0, substitution matrix = Blosum62, substitution probability threshold T = 0.9) with the ones studied in [33] (FDC, AAC, and Blast coupled each one with the nearest neighbor algorithm NN). Comparative results are reported in table 8. We can notice that the worst results were obtained with the AAC method. Indeed, the obtained results were below the MAV 46.7%. Blast arrived at better results, but the accuracy was not very high. In fact, an analysis of the Protein Data Bank (PDB) [50], where the protein structures are deposited, reveals that proteins with more than 30% pairwise sequence identity have similar 3D structures [51]. But in our case we process a dataset with a sequence identity of 25%. The FDC method seems to be promising since it allowed reaching an accuracy of 75.2%. But our method was quite better and enabled to reach the highest accuracy rates among the mentioned methods and also coupled with the same classifier i.e., NN algorithm (77%).

**Table 8:** Comparison with results reported in (Yu et al., 2006) for DS3

Methods	Accuracy %	Correctly classified sequences
DDSM & C4.5	79.2	568
DDSM & SVM	78.9	588
DDSM & NB	59.4	434
DDSM & NN	77	564
FDC & NN	75.2	539
AAC & NN	41.4	297
Blast-based	69.6	499

If we look for better classification systems we can consider the combinations (DDSM & C4.5) or (DDSM & SVM). In addition higher accuracy can be obtained by using the combination (DDSM & SVM) and the matrix Pam30 which enabled to reach an accuracy of 82% (table 8). This indicates that SVM coupled with our encoding method DDSM represent an efficient system for protein classification.

In the same way, the use of the same dataset (DS5) and the same validation technique (leave-one-out) as in [27,28] allowed us to compare our method with these two works as well as six others [34-39]. In these studies, variants of the amino acid composition AAC have been proposed to encode protein sequences and then coupled with a classifier to predict the protein structural classes. These works are based in the assumption that there is a strong correlation between the AAC and the structural class of a protein. In table 9, we report the results obtained by our method (DDSM with default parameter values: α = 0, β = 0, substitution matrix = Blosum62, substitution probability threshold T = 0.9) coupled with C4.5, SVM, NB and NN as well as the results of the related works (in table 9, AAC_x _means the AAC variant presented in the paper x). We can claim that our encoding method generally outperforms any AAC encoding method proposed by the above-mentioned works. In [27], authors coupled three kinds of AAC with SVM i.e., (AAC & SVM), (pair-coupled AAC & SVM) and (PseAAC & SVM). In the best case, they reached an accuracy of 80.5%, whereas the combinations (DDSM & SVM) and (DDSM & NB) allowed reaching respectively 82.3% and 85.9% of accuracy. To enhance their results, authors in [27] proposed a fusion network that combines the results obtained by the three proposed combinations and they arrived at an accuracy of 87.7%. Although, this result is slightly superior to ours, it does not mean that their encoding method outperforms DDSM. Indeed, the improvement of their results comes from the fusion network classifier and not from the AAC variants they use. Moreover, in most of these related works [27,28,34-39], authors perform a fine-tuning to look for the classifier parameter values allowing to get the best results, whereas we just use the default parameter values of both our encoding method and the classifiers as found in WEKA [32]. This fine tuning allowed to reach competitive accuracies which is the case of the combination (AAC & LogitBoost) [38]. We believe that we can also reach higher accuracies if we perform a fine-tuning of the parameters of our method and the classifiers. But, we chose to just use the default parameter values to make it easier for users who may have no prior knowledge on what these parameters mean or how to specify them.

**Table 9:** Comparison with results reported in (Chen et al., 2006) and (Zhou, 1998) for DS5

Methods	Accuracy %	Correctly classified sequences
DDSM & C4.5	73.3	203
DDSM & SVM	82.3	228
DDSM & NB	85.9	238
DDSM & NN	78	216
Blast-based	78.3	220
AAC[27] & SVM [27]	80.5	223
pair-coupled AAC[27] & SVM [27]	77.6	215
PseAAC[27] & SVM [27]	80.5	223
SVM fusion [27]	87.7	243
AAC[28] & Component coupled [28]	79.1	219
AAC[34] & City-block distance [34]	59.9	166
AAC[35] & Euclidean distance [35]	55.2	153
AAC[36] & Neural network [36]	74.7	206
AAC[37] & SVM [37]	79.4	219
AAC[38] & LogitBoost [38]	84.1	233
AAC[39] & Rough Sets [39]	79.4	219

## Competing interests

The authors declare that they have no competing interests.

## Authors' contributions

RS participated in the design of the DDSM method, implemented the programs, selected data, carried out the experimental study, performed the statistical analysis and drafted and revised the manuscript. MM conceived the DD method, participated in DDSM design, participated in the data selection and revised the manuscript. EMN conceived the DDSM method, provided assistance in the statistical analysis and revised the manuscript. All authors read and approved the final manuscript.

## Supplementary Material

Additional file 1 **Software, 185 K**. SeqCod.zip contains programs which are also available upon request from the authors or from http://www.cril.univ-artois.fr/~mephu/SeqCod and http://www.isima.fr/~mephu/FILES/SeqCod/. Click here for file

Additional file 2 **Experimental data, 328 K**. Experimental_data.zip comprises all datasets with their classification files. Click here for file
