# Supplementary material for: Protein sequences classification by means of feature extraction with substitution matrices
Source: BMC Bioinformatics. 2010 Apr 8;11:175. doi: 10.1186/1471-2105-11-175 (PMC2868007; doi:10.1186/1471-2105-11-175)
Supplement: Additional file 1 — Software, 185 K. SeqCod.zip contains programs which are also available upon request from the authors or from http://www.cril.univ-artois.fr/~mephu/SeqCod and http://www.isima.fr/~mephu/FILES/SeqCod/. [file 1471-2105-11-175-S1.ZIP › SeqCod/SeqCod_read_me.pdf]

Relational and Binary Coding Methods Library for Biological Sequences

1 Description

This library comprises methods to re-encode biological sequences (DNA and protein) into relational or binary formats. Methods have been developed in C language and can be called by the following interface:

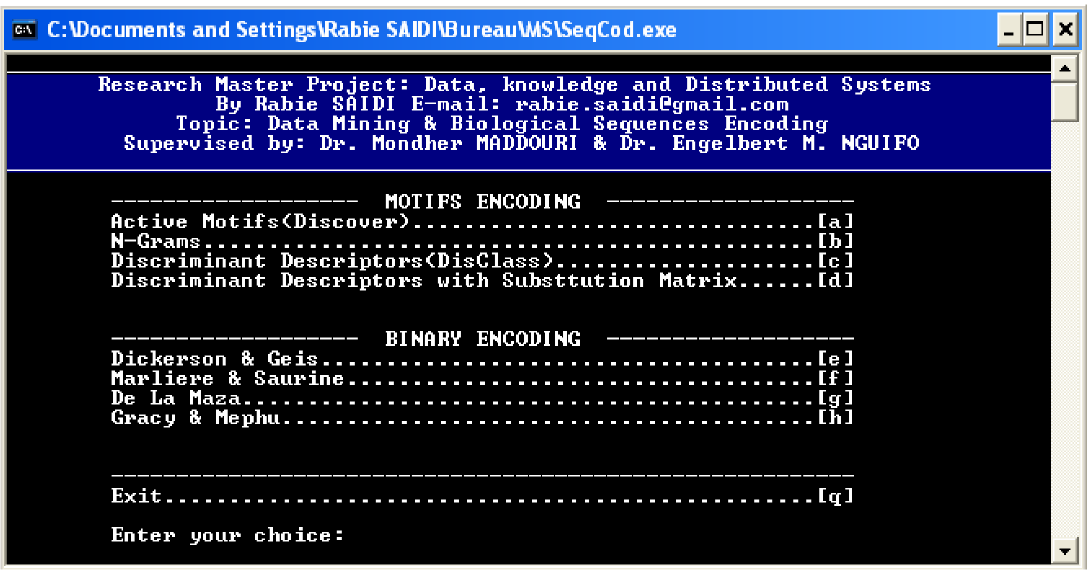

Figure 1. Main menu.

The menu consists of 2 sections:

1.1 Motifs based encoding methods:

- Active Motifs [Wang et al., 1994]
- N-Grams [Leslie et al., 2002]
- Discriminant Descriptors [Maddouri and Elloumi, 2004]
- Discriminant Descriptors with Substitution Matrices [Saidi et al., 2007]

The generated files by these methods are under relational format, namely ARFF format (Attribute Relation File Format) used by the workbench Weka [Witten and Eibe 2005].

1.2 Binary encoding methods:

- Dickerson & Geis [Dickerson et al, 1969]
- Marliere & Saurine [Fu, 2001]
- De La Maza [De la Maza, 1994]
- Gracy & Mephu [Nguifo, 1993]

The generated files by these methods comprise binary sequences.

2 How to use

- Two files are needed i.e. SeqCod.exe and DLL_SeqCod.dll, to run the application.
- Sequences file(s) in fasta format
- Classification file(s) describing the sequences file (for methods based on motifs)
- Select a method to apply

2.1 Fasta format

```
>
MTSIFHFAIIFMLILQIRIQLSEESEFLVDRSKNGLIHVPKDLSQKTTILNISQNYISEL
LRILIISHNRIQYLDISVFKFNQELEYLDLSHNKLVKISCHPTVNLKHLDLSFNAFDALP
LKFLGLSTTHLEKSSVLPIAHLNISKVLLVLGETYGEKEDPEGLQDFNTESLHIVFPTNK
KTVANLELSNIKCVLEDNKCSYFLSILAKLQTNPKLSSLTLNNIETTWNSFIRILQLVWH
VKLQGQLDFRDFDYSGTSLKALSIHQVVSDVFGFPQSYIYEIFSNMNIKNFTVSGTRMVH
LHLDFSNNLLTDTVFENCGHLTELET
>
MPATSSIITIIAVAACLLLLVADAHAQQQCNWQYGLTTMDIRCSVRALESGTGTPLDLQV
CSQELLHASELAPGLFRQLQKLSELRIDACKLQRVPPNAFEGLMSLKRLTLESHNAVWGP
FQGLKELSELHLGDNNIRQLPEGVWCSMPSLQLLNLTQNRIRSAEFLGFSEKLCAGSALS
ELQTLDVSFNELRSLPDAWGASRLRRLQTLSLQHNNISTLAPNALAGLSSLRVLNISYNH
GNKELRELHLQGNDLYELPKGLLHRLEQLLVLDLSGNQLTSHHVDNSTFAGLIRLIVLNL
```

Figure 2. FASTA format. Each sequence starts by '>'.

2.2 Classification file

```
#classes number
2
#classes names
TLRH
TLRNH
#classes instances
TLRH
TLRNH
TLRNH
TLRNH
```

Figure 3. Cassification file

The classification file above descibe a fasta file containing 4 biological sequences belonging to 2 classes: the 1st belongs to the « TLRH » class and the 3 others belong to the «TLRNH ».
To make the generation of such file easier, an application has been developed: **ClassFileGen.exe**.

2.3 Motifs encoding methods

2.3.1 Common parameters

- Enter the **FASTA file name** (don't forget file extension, e.g. seq_file.txt),
- Enter the **classification file name** (don't forget file extension, e.g. seq_file_class.txt),
- If there exists a **test file** then enter its name and the name of its **classification file**,
- When all parameters are set, enter the **name of the output file** (don't forget file extension, e.g. out_file.arff).

2.3.2 Active Motifs

- Select **motif shape**: *X* (for simple motifs, e.g. RSMT) or *X*Y* (for compound motifs: with gap, e.g. RSMT*VFF),
- Set the **minimum length** of motifs,
- Set the **minimum occurrence number** of motifs,
- Set the **number of allowed mutations** (e.g. if number of allowed mutations = 1 then the motifs RSMT and RSVT are considered the same).

2.3.3 N-Grams

- Enter the **length** of motifs (it is a fixed length: 3 by default → 3-grams).

2.3.4 Discriminant Descriptors

- Set **alpha threshold** of motifs: minimum occurrence rate of motifs within a defined sequence family *F* (e.g: 0.9),
- Set **beta threshold** of motifs: maximum occurrence rate of motifs within all sequence families excluding *F*, i.e. other families than the family *F* (e.g: 0.08),
- E.g. "RSMT" is a considered as motif of a family *F* iff it occurs in at least 90% of the sequences of *F* and at most 8% of the database sequences excluding *F*, i.e. other families than the family *F*.

2.35 Discriminant Descriptors with Substitution Matrices

- Set **alpha and beta thresholds** (as in Discriminant Descriptors section),
- Select the **substitution matrix number** (e.g: 2 for Blosum62),
- Set the **similarity score threshold** (or **substitution probability**): We consider that a motif *M* substitutes a motif *M'* if their substitution probability is higher than a given threshold.

2.3 Motifs encoding methods

- Enter the **FASTA file name** (don't forget file extension, e.g. seq_file.txt),
- Enter the **name of the output file**.

References

De la Maza M. Generate, test and explain:synthesizing regularity exposing attributes in large protein databases. In 27th Hawain Intl conf on System Science (HICSS) Hawaï, pages 123-129, 1994.

Dickerson R.E. & I. Geis. The Structure and Actions of Proteins. Harper and Row Publishers, New York - USA, 1969.

Fu H. Intelligence Artificielle et codage de séquences de protéines. Rapport de Master. CRIL, Lens, Juillet 2001.

Leslie C., Eskin E. and W. S. Noble. (2002) The spectrum kernel: a string kernel for svm protein classification. *Pacific Symposium on Biocomputing (PSB)*, 564–575.

Maddouri M. and Elloumi M. (2004) Encoding of primary structures of biological macromolecules within a data mining perspective. *Journal of Computer Science and Technology (JCST)*, 19(1):78-88. Allerton Press. USA.

Nguifo E. M. Concevoir une abstraction à partir de ressemblances. Thèse de doctorat d'université. Université de Montpellier II, mai 1993.

Saidi R., Maddouri M. & Mephu Nguifo E. (2007) Biological Sequences Encoding for Supervised Classification. *Intl. conf. on BioInformatics Research and Development (BIRD), TU Berlin, March 12-14, Springer-Verlag*, LNBI 4414, 224-238.

Wang J. T. L., Marr T. G., Shasha D., Shapiro B. A. and Chirn G. W. (1994). Discovering active motifs in sets of related protein sequences and using them for classification. *Nucleic Acids Research*, 22(14): 2769-2775.

Witten I. H. and Eibe F. (2005). Data Mining: Practical machine learning tools and techniques, 2nd Edition. Morgan Kaufmann, San Francisco.

Leslie C., Eskin E. and W. S. Noble. (2002) The spectrum kernel: a string kernel for svm protein classification. Pacific Symposium on Biocomputing (PSB), 564–575.
